# Three-Dimensional CT for Quantification of Longitudinal Lung and Pneumonia Variations in COVID-19 Patients

**DOI:** 10.3389/fmed.2021.643917

**Published:** 2021-03-25

**Authors:** Qiuying Chen, Lv Chen, Shuyi Liu, Luyan Chen, Minmin Li, Zhuozhi Chen, Jingjing You, Bin Zhang, Shuixing Zhang

**Affiliations:** Department of Radiology, The First Affiliated Hospital of Jinan University, Guangzhou, China

**Keywords:** coronavirus disease 2019, computed tomography, CT score, CAD system, low attenuation areas

## Abstract

**Objectives:** Visual chest CT is subjective with interobserver variability. We aimed to quantify the dynamic changes of lung and pneumonia on three-dimensional CT (3D-CT) images in coronavirus disease 2019 (COVID-19) patients during hospitalization.

**Methods:** A total of 110 laboratory-confirmed COVID-19 patients who underwent chest CT from January 3 to February 29, 2020 were retrospectively reviewed. Pneumonia lesions were classified as four stages: early, progressive, peak, and absorption stages on chest CT. A computer-aided diagnostic (CAD) system calculated the total lung volume (TLV), the percentage of low attenuation areas (LAA%), the volume of pneumonia, the volume of ground-glass opacities (GGO), the volume of consolidation plus the GGO/consolidation ratio. The CT score was visually assessed by radiologists. Comparisons of lung and pneumonia parameters among the four stages were performed by one-way ANOVA with *post-hoc* tests. The relationship between the CT score and the volume of pneumonia, and between LAA% and the volume of pneumonia in four stages was assessed by Spearman's rank correlation analysis.

**Results:** A total of 534 chest CT scans were performed with a median interval of 4 days. TLV, LAA%, and the GGO/consolidation ratio were significantly decreased, while the volume of pneumonia, GGO, and consolidation were significantly increased in the progressive and peak stages (for all, *P* < 0.05). The CT score was significantly correlated with the pneumonia volume in the four stages (*r* = 0.731, 0.761, 0.715, and 0.669, respectively, *P* < 0.001).

**Conclusion:** 3D-CT could be used as a useful quantification method in monitoring the dynamic changes of COVID-19 pneumonia.

## Introduction

Rapid and accurate diagnosis is urgently needed for the coronavirus disease 2019 (COVID-19) pandemic. In addition to real-time fluorescence polymerase chain reaction (RT-PCR), chest CT has been extensively used as a convenient and highly sensitive tool for screening, diagnosis, and follow-up of COVID-19 since its outbreak (1). Bilateral peripheral ground-glass opacities (GGO) with or without consolidation are the most common lung appearance of COVID-19 (2). Some recent studies have depicted typical CT manifestations of COVID-19 pneumonia during disease course into the early, progressive, peak, and absorption stages (3–6). Semiquantitative CT score may reflect the severity of the disease, which is a simple marker in daily practice (7). However, radiologist-interpreted chest CT is limited by larger interobserver variability, time-consuming, and inefficient.

The serial lung changes in addition to pneumonia lesions were not taken into account in the follow-up plan of COVID-19 patients. The percentage of low-attenuation lung tissues can be used to describe the severities of the lung disease; low attenuation areas (LAAs) represent individual areas of emphysematous destruction (8). Previous studies failed to consider the coexisting chronic pulmonary abnormalities in COVID-19 patients, for instance, emphysema and interstitial lung diseases (7). Colombi et al. found that well-aerated lung volume on initial CT may indicate the severity of disease and was a predictor of intensive care unit (ICU) admission or death in patients with COVID-19 pneumonia (9). To date, no lung functional data are available for COVID-19 patients during hospitalization except for Mo et al. who found the impairment of lung volume in COVID-19 patients prior to discharge, especially in severe cases (10). Previous studies have shown that some recovered patients with other coronavirus pneumonias [e.g., severe acute respiratory syndrome (SARS) and Middle East respiratory syndrome (MERS)] may be left with persistently damaged lungs, which could last for months or even years (11–14). It would be of clinical importance to monitor the dynamic change of lung in COVID-19 patients after treatment and might determine the necessity for subsequent pulmonary function test and pulmonary rehabilitation.

In this study, we aimed to quantify the serial changes of lung and pneumonia on three-dimensional CT (3D-CT) in COVID-19 patients using a computer-aided diagnostic (CAD) system, which provides the imaging means for medical professionals to rapidly and accurately evaluate disease severity and treatment response of COVID-19 patients. This may play an important role in disease monitoring, early intervention, and determination of the timing of admission.

## Materials and Methods

### Study Population

**Figure 1:**
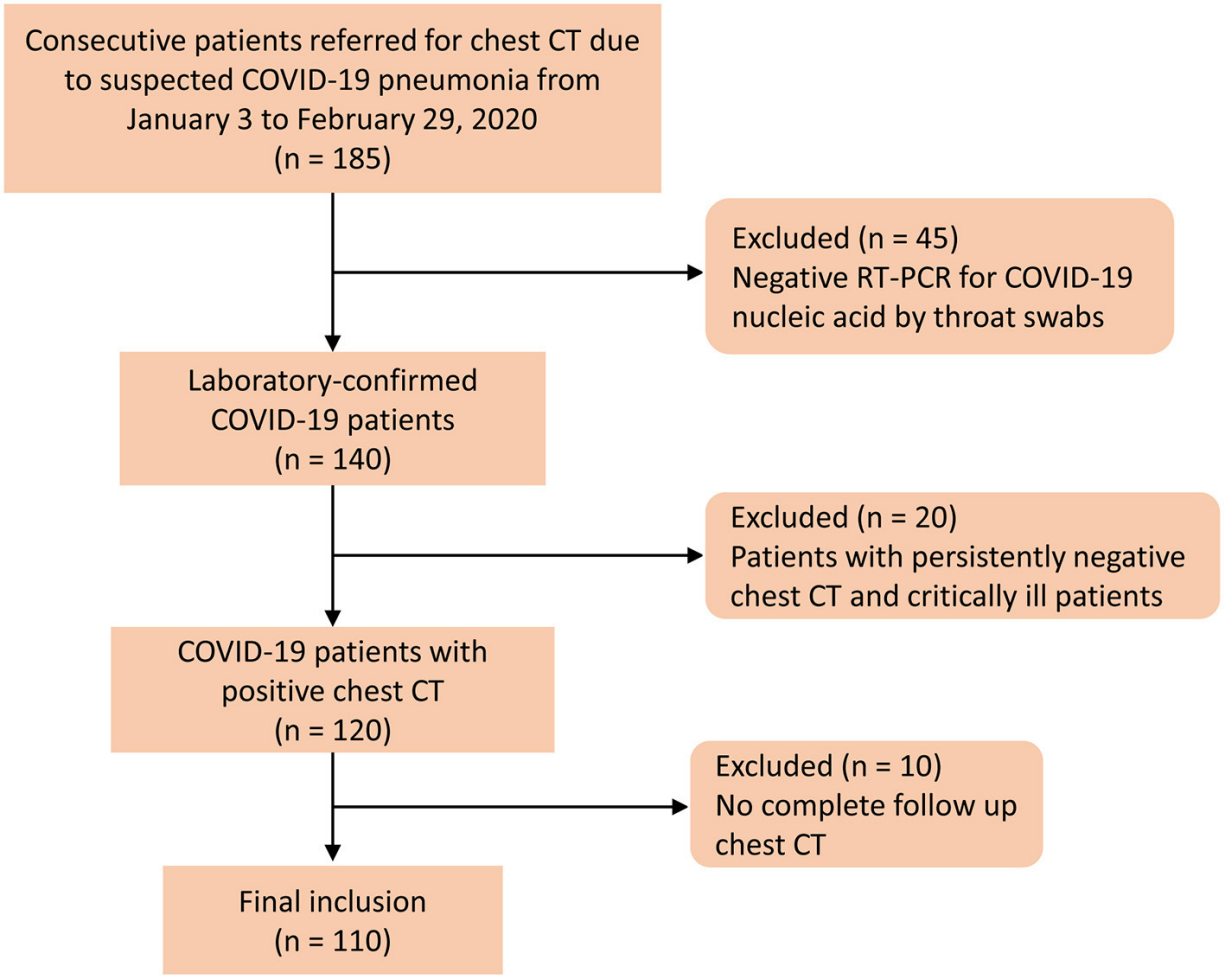
Flow diagram of the patient selection process. Negative RT-PCR was defined as at least two repetitive negative RT-PCR test results, separated by at least 1 day.

The retrospective study was approved by an ethics committee of our institution, and the informed consent was waived. A total of 185 patients were admitted to a designated hospital due to suspicion of COVID-19 between January 3 and February 29, 2020. The inclusion criteria were as follows: (1) patients were positive for COVID-19 through RT-PCR for COVID-19 nucleic acid by throat swabs (at least two samples were taken, at least 24 h apart); (2) patients had at least one positive chest CT during hospitalization; and (3) patients had follow-up chest CT. The disease severity of COVID-19 was categorized into mild, moderate, severe, and critical illness on the basis of the newest COVID-19 guidelines released by the National Health Commission of China (15). Patients had persistently negative chest CT, and critically ill patients who underwent X-rays were excluded. Figure 1 shows the patients' enrollment flowchart. Finally, 110 COVID-19 patients were included for analysis.

### CT Examinations

All patients underwent unenhanced chest CT scans by a Siemens Emotion 16 scanner (Siemens Healthineers; Erlangen, Germany), a CT 64 scanner (GE Medical System), or an ICT 128 scanner (Philips Healthcare, Netherlands). No contrast agent was administered. CT acquisition of the GE 64 scanner was executed as follows: tube voltage, 120 kV; tube current, 260 mAs; pitch, 0.984; and slice thickness reconstructions of 0.625 mm. CT acquisition of the Siemens 16 scanner was executed as follows: tube voltage, 130 kV; automatic tube current; pitch, 1.5; and slice thickness reconstructions of 1.0 or 0.6 mm. CT acquisition of the ICT 128 scanner was executed as follows: tube voltage, 120 kV; automatic tube current; pitch, 0.7; collimation, 0.625 mm and slice thickness reconstructions of 1.0 or 0.67 mm.

### Quantitative CT Analysis

According to a previous study (3), early, progressive, peak, and absorption stages on chest CT were defined as 0–4, 5–8, 9–13, and ≥14 days after the onset of the initial symptoms, respectively. A CT scoring system was used to assess the involvement area/degree of pneumonia for each lung lobe: 0 for 0%; 1 for 1–25%; 2 for 26–50%; 3 for 51–75%; and 4 for 76–100% (2). A CT score (range, 0–20) was assigned by summarizing the total scores for the five lobes. All the chest CT images were reviewed independently by two radiologists (with more than 10 years of experience), who were blinded to clinical and laboratory results. Any discrepancy was resolved by a consensus viewing.

**Figure 2:**
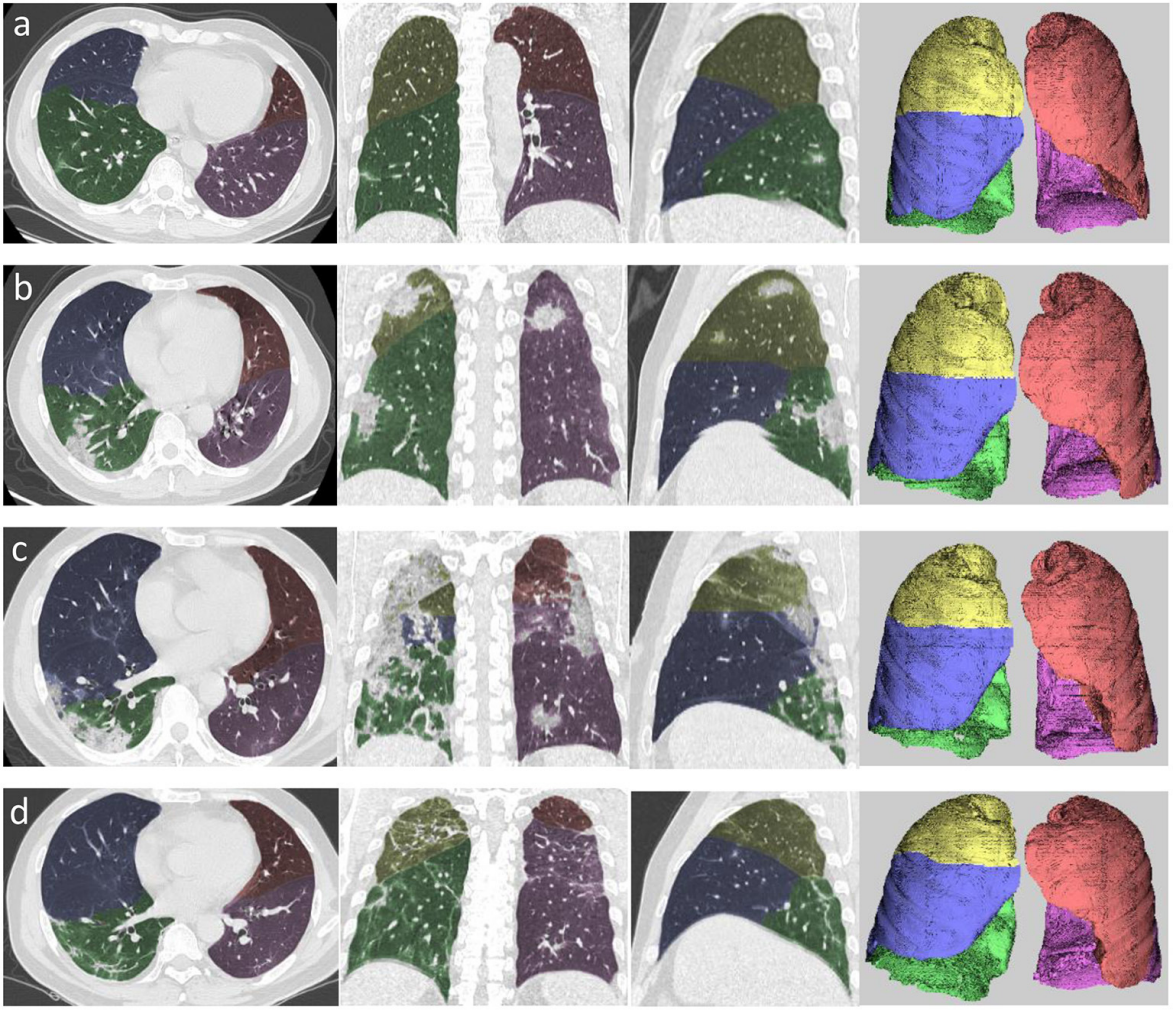
Example of segmentation using automated computer-aided diagnosis in the four stages of COVID-19 pneumonia. **(a)** Axial, coronal, and sagittal multiplanar reconstruction views and volume rendering in the early stage. **(b)** Axial, coronal, and sagittal multiplanar reconstruction views and volume rendering (left to right) in the progressive stage. **(c)** Axial, coronal, and sagittal multiplanar reconstruction views and volume rendering (left to right) in the peak stage. **(d)** Axial, coronal, and sagittal multiplanar reconstruction views and volume rendering (left to right) in the absorption stage. Yellow area denotes right upper lobe, blue area denotes right middle lobe, green area denotes right lower lobe, orange area denotes left upper lobe, and pink area denotes left lower lobe.

The chest CT images were transferred to a workstation (Synapse Image Intelligence™ Vincent version 4.4; Fujifilm Medical Systems, Tokyo, Japan). This workstation implemented a lobar CAD system that was showed to accurately measure lobar volumes (16). This system automatically extracted both lungs, recognized lobar bronchi, and determined the locations of fissures (Figure 2). Subsequently, the CAD system semi-automatically extracted pneumonia regions after simply dragging both ends of the infected regions. Two cardiothoracic radiologists checked the lesion segmentation by CAD and made manual corrections by delineating fissures and contour of infected regions when the CAD system failed to properly identify these.

**Figure 3:**
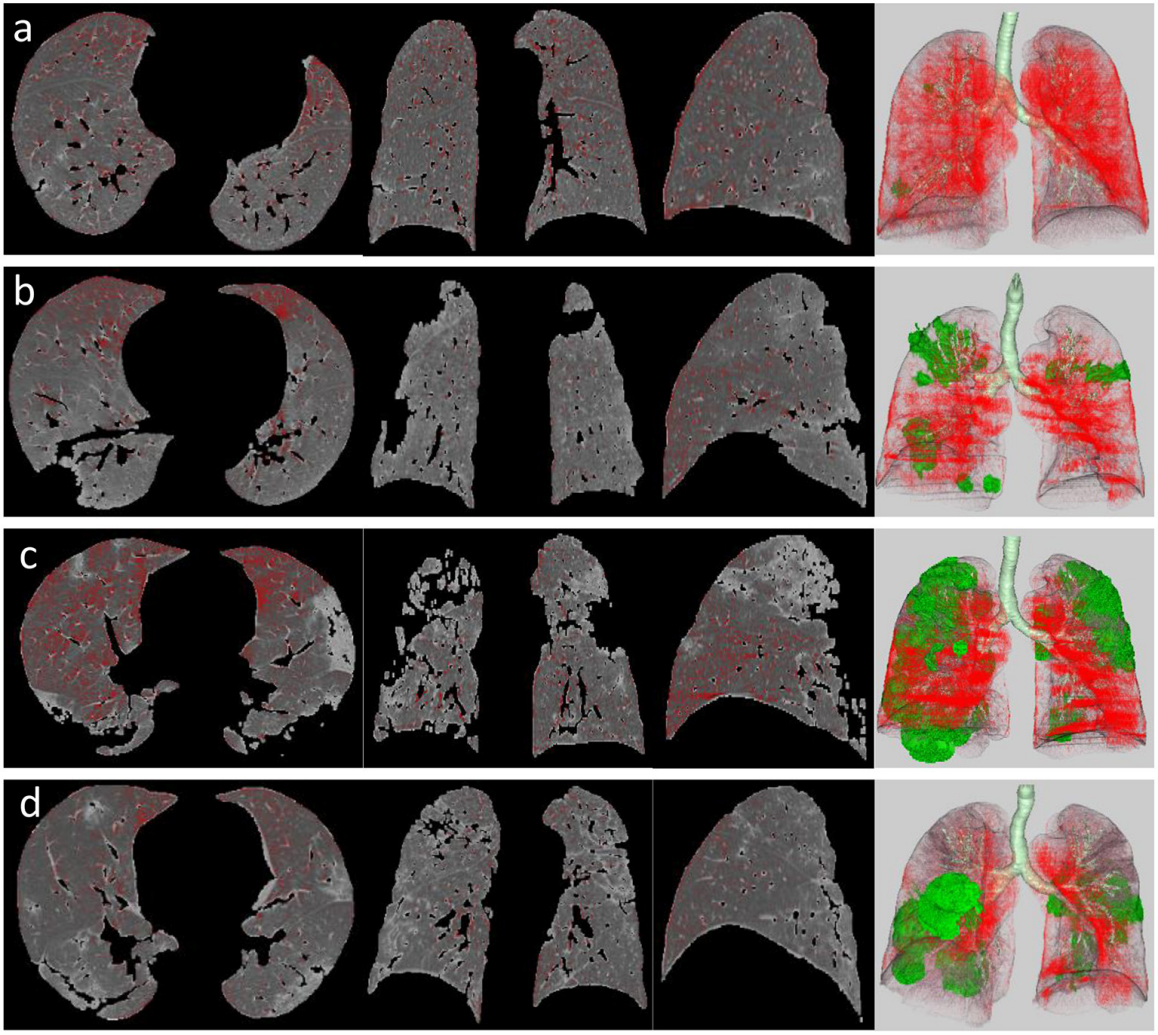
LAA views of the four stages in a patient with COVID-19 pneumonia, which was defined as the lung field area with attenuation values <-950 HU of threshold (red areas). It shows axial, coronal, and sagittal two-dimensional (2D) displays and three-dimensional (3D) image (left to right) of LAA distribution in the early stage **(a)**, progressive stage **(b)**, peak stage **(c)**, and absorption stage **(d)**. Green area on 3D image represents pneumonia lesions.

**Figure 4:**
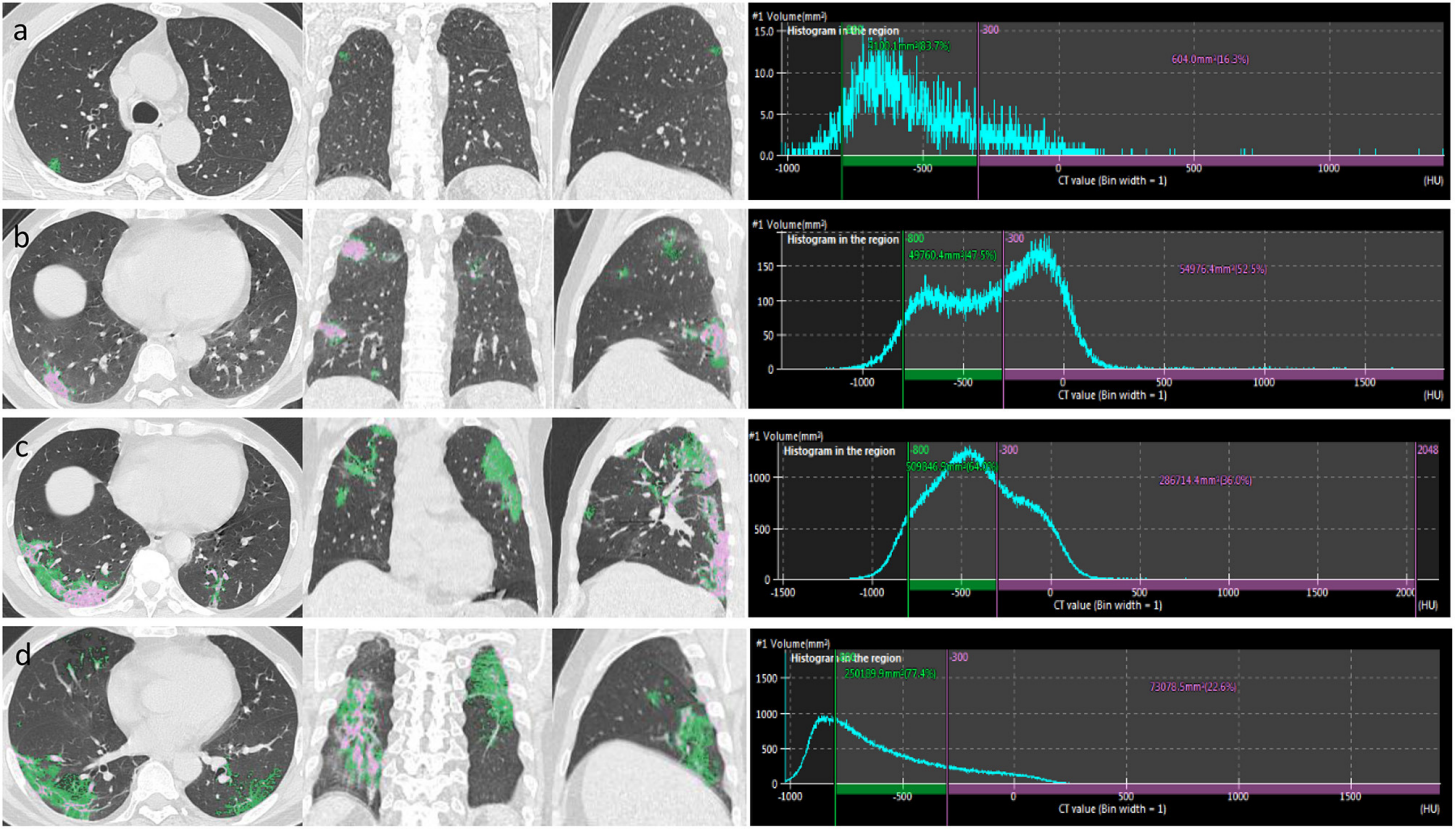
After the segmentation of infected regions, automatic extraction of GGO and consolidation was performed in a patient with COVID-19 pneumonia. By thresholding on CT values in the pneumonia lesions, GGO (green area) and consolidation (pink area) were identified with the value range of −800 to −300 and >-300 HU, respectively. The figure shows axial, coronal, and sagittal multiplanar reconstruction views (left to right) of GGO and consolidation in the early stage **(a)**, progressive stage **(b)**, peak stage **(c)**, and absorption stage **(d)**.

A quantitative analysis procedure was performed based on the segmentation results. The lung field area with attenuation values < −950 Hounsfield Unit (HU) of thresholds was considered as LAAs (Figure 3). The proportion of LAAs (LAA%) for total lung volume was calculated automatically. By thresholding on CT values in the pneumonia lesions, GGO and consolidation were identified with the value range of −800 to −300 HU and −300 to 50 HU, respectively (17) (Figure 4). The volume and percentage of GGO and consolidation were calculated, accordingly. The ratio of GGO to consolidation was also computed.

### Statistical Analysis

Total lung volume, LAA%, the volume of pneumonia, the volume of consolidation, the percentage of GGO, the percentage of consolidation, and the GGO/consolidation ratio obtained at the four stages were compared using one-way ANOVA with *post-hoc* LSD multiple comparison tests at a level of significance *P* < 0.05. Spearman's rank correlation analysis was used to assess the correlation between the CT score and the volume of pneumonia. All statistical analyses were performed using the SPSS statistical software package (version 23, SPSS Inc., Chicago, IL, USA).

## Results

### Clinical Characteristics

The mean age of the 110 patients was 45.2 ± 12.8 years (range, 14–80 years) and 65 (59.1%) were males. A total of 100 patients (90.9%) had moderate illness, and 10 patients (9.1%) had severe illness. A total of 534 chest CT scans were conducted with a median of 4 scans (interquartile range, 3–5) per patient. The total CT scans for the early, progressive, peak, and absorption stages were 91 (17.0%), 188 (35.2%), 39 (7.3%), and 216 (40.4%), respectively. A total of 62 (56.4%), 89 (80.9%), 18 (16.4%), and 95 (86.4%) patients experienced early, progressive, peak, and absorption stages, respectively.

### Comparison of Lung and Pneumonia Parameters Generated by the CAD System

Table 1 shows the comparison of lung and pneumonia parameters among the four stages. As compared with the CT score of early stage, CT scores of the progressive stage (7.6 ± 3.2 vs. 3.2 ± 2.1, *P* < 0.001) and the peak stage (10.3 ± 4.4 vs. 3.2 ± 2.1, *P* < 0.001) were significantly higher. Table 1 shows that the total lung volume significantly decreased in the progressive stage (3.5 L ± 1.1 vs. 4.2 L ± 1.2, *P* < 0.001) and the peak stage (3.1 L ± 0.9 vs. 4.2 L ± 1.2, *P* < 0.001) and then recovered in the absorption stage (3.8 L ± 1.1 vs. 4.2 L ± 1.2, *P* = 0.008). The bilateral lower lobes were the most infected. The volume of pneumonia was significantly increased in the progressive stage (431.5 ± 409.3 cm^3^ vs. 73.8 ± 96.0 cm^3^, *P* < 0.001) and the peak stage (568.1 ± 470.6 cm^3^ vs. 73.8 ± 96.0 cm^3^, *P* < 0.001) and then decreased in the absorption stage (291.0 ± 404.6 cm^3^ vs. 73.8 ± 96.0 cm^3^, *P* < 0.001). The mean time to peak of the pneumonia volume was 5.2 ± 4.8 days. In the early stage, 22 patients (20%) had LAA% of <5%, 78 patients (70.9%) had LAA% of 5–24%, and 10 patients (9.1%) had LAA% of 25–49%. LAA% decreased in the progressive stage (10.1 ± 6.6% vs. 13.7 ± 8.0%, *P* < 0.001), the peak stage (10.3 ± 5.7% vs. 13.7 ± 8.0%, *P* < 0.01), and the absorption stage (9.8 ± 6.1% vs. 13.7 ± 8.0%, *P* < 0.001). GGO volume increased in the progressive stage (230.6 cm^3^ ± 231.0 vs 41.4 cm^3^ ± 59.2, *P* < 0.001), peak stage (328.3 cm^3^ ± 299.0 vs 41.4 cm^3^ ± 59.2, *P* < 0.001), but decreased in the absorption stage (155.4 cm^3^ ± 204.7 vs 41.4 cm^3^ ± 59.2, *P* < 0.001). Consolidation volume increased in the progressive stage (125.6 cm^3^ ± 122.1 vs 21.6 cm^3^ ± 30.9, *P* < 0.001), peak stage (157.7 cm^3^ ± 124.4 vs 21.6 cm^3^ ± 30.9, *P* < 0.001), but decreased in the absorption stage (49.4 cm^3^ ± 71.5 vs 21.6 cm^3^ ± 30.9, *P* < 0.05). GGO: consolidation ratio was significantly reduced in the progressive stage (3.0 ± 4.0 vs 31.2 ± 160.5, *P* < 0.01), peak stage (2.6 ± 2.7 vs 31.2 ± 160.5, *P* < 0.05) but increased in the absorption stage (9.5 ± 36.2 vs 31.2 ± 160.5, *P* < 0.05).

**Table 1:** Comparison of lung and pneumonia parameters generated by the CAD system among the four stages.

**Parameters**	**Early stage (*n* = 91)**	**Progressive stage (*n* = 188)**	**Peak stage (*n* = 39)**	**Absorption stage (*n* = 216)**	***P*-value^#^**
Total lung volume, L	4.2 (1.2)	3.5 (1.1)^+++^	3.1 (0.9)^+++^	3.8 (1.1)^++^	<0.001
Volume of left lung, L	1.9 (0.6)	1.7 (0.5)^+++^	1.5 (0.5)^+++^	1.8 (0.5)^+^	<0.001
Volume of LUL, L	1.1 (0.3)	1.0 (0.3)	0.9 (0.3)^+^	1.0 (0.3)	0.102
Volume of LLL, L	0.9 (0.3)	0.7 (0.3)^+++^	0.6 (0.3)^+++^	0.8 (0.3)^+++^	<0.001
Volume of right lung, L	2.2 (0.6)	1.9 (0.6)^+++^	1.6 (0.4)^+++^	2.0 (0.6)^++^	0.013
Volume of RUL, L	0.9 (0.3)	0.9 (0.3)	0.8 (0.3)^+++^	0.9 (0.3)	<0.001
Volume of RML, L	0.4 (0.2)	0.4 (0.2)	0.4 (0.2)	0.4 (0.2)	0.957
Volume of RLL, L	0.9 (0.4)	0.7 (0.4)^+++^	0.5 (0.3)^+++^	0.8 (0.3)^+++^	<0.001
LAA% of total lung	13.7 (8.0)	10.1 (6.6)^+++^	10.3 (5.7)^++^	9.8 (6.1)^+++^	<0.001
LAA% of left lung	14.0 (8.2)	10.5 (6.8)^+++^	10.6 (5.6)^++^	10.3 (6.3)^+++^	<0.001
LAA% of LUL	16.6 (8.6)	13.1 (7.8)	12.2 (5.9)	15.1 (33.8)	0.586
LAA% of LLL	11.6 (8.0)	7.5 (6.2)^+++^	7.6 (5.3)^+++^	7.0 (5.5)^+++^	<0.001
LAA% of right lung	13.4 (8.0)	9.8 (6.4)^+++^	10.0 (5.9)^++^	9.3 (5.9)^+++^	<0.001
LAA% of RUL	14.9 (8.3)	11.4 (7.1)^+++^	10.4 (5.6)^+++^	11.0 (6.6)^+++^	<0.001
LAA% of RML	16.3 (8.6)	12.7 (7.9)^+++^	12.7 (7.3)^+^	12.0 (7.1)^+++^	<0.001
LAA% of RLL	11.3 (8.1)	6.8 (6.1)^+++^	7.2 (5.5)^+++^	6.3 (5.4)^+++^	<0.001
Volume of pneumonia, cm^3^	73.8 (96.0)	431.5 (409.3)^+++^	568.1 (470.6)^+++^	291.0 (404.6)^+++^	<0.001
Volume of GGO, cm^3^	41.4 (59.2)	230.6 (231.0)^+++^	328.3 (299.0)^+++^	155.4 (204.7)^+++^	<0.001
Volume of consolidation, cm^3^	21.6 (30.9)	125.6 (122.1)^+++^	157.7 (124.4)^+++^	49.4 (71.5)^+^	<0.001
GGO: consolidation ratio	31.2 (160.5)	3.0 (4.0)^++^	2.6 (2.7)^+^	9.5 (36.2)^+^	0.015

*Values are expressed as mean (SD)*.

# *One-way ANOVA with post-hoc LSD multiple comparison tests was used to test the differences between data at early, progressive, peak, and absorption stages. The early stage was used as a reference*.

+ *P < 0.05*,

++ P < 0.01, and

+++ *P < 0.001. LUL, left lower lobe; LUL, left upper lobe; RLL, right lower lobe; RML, right middle lobe; RUL, right upper lobe; LAA%, percentage of low attenuation areas; GGO, ground-glass opacities*.

### Relationship Between CT Score and Volume of Pneumonia

**Figure 5:**
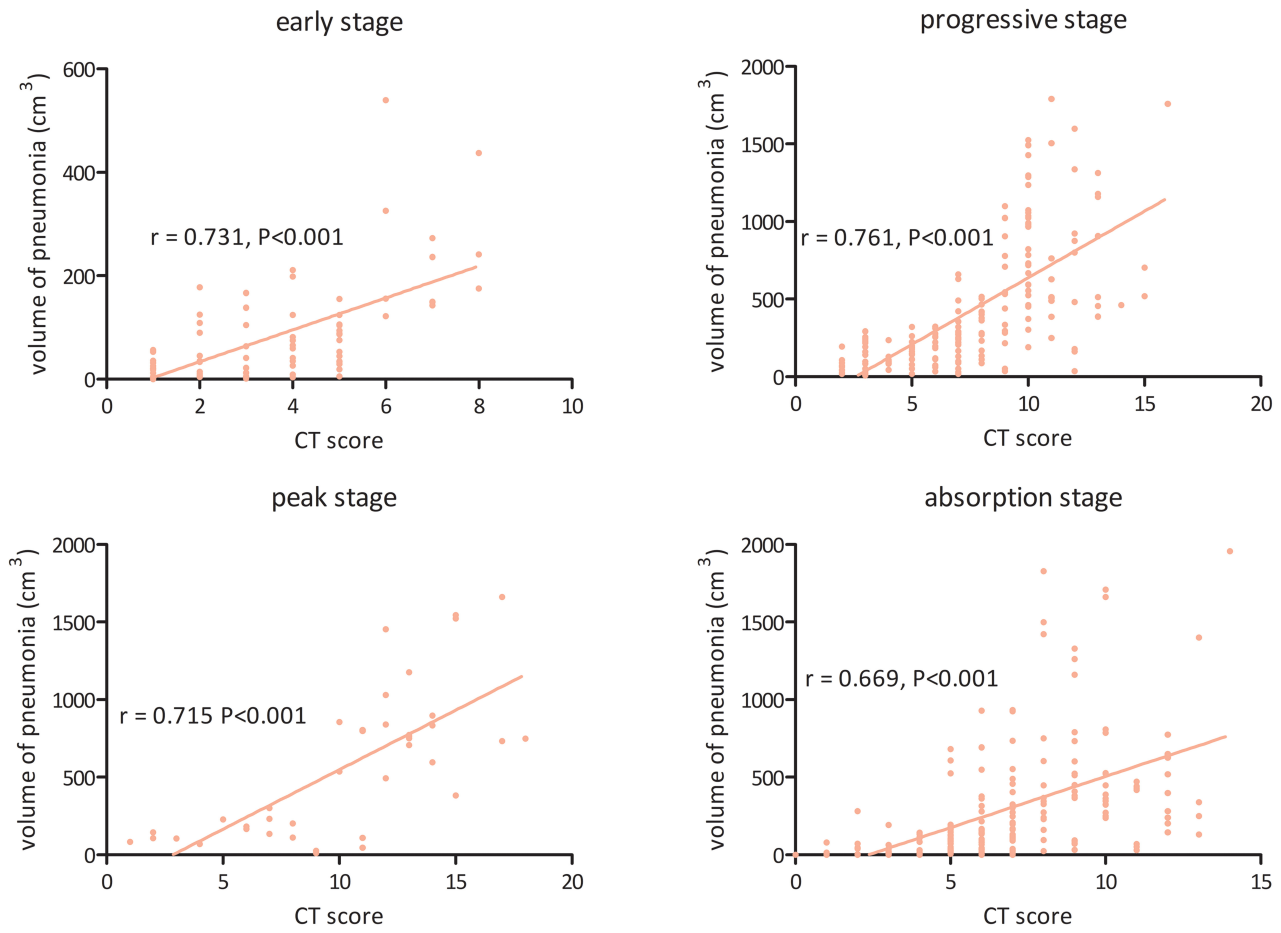
The association between visual CT score and volume of pneumonia measured by the computer-aided diagnosis system in the four stages of COVID-19 pneumonia.

Figure 5 shows the association between the visual CT score and the volume of pneumonia measured by the CAD system in the four stages. The CT score was found to be significantly correlated with the volume of pneumonia in the early stage (*r* = 0.731, 95% CI: 0.614–0.816, *P* < 0.001), the progressive stage (*r* = 0.761, 95% CI: 0.692–0.817, *P* < 0.001), the peak stage (*r* = 0.715, 95% CI: 0.508–0.844, *P* < 0.001), and the absorption stage (*r* = 0.669, 95% CI: 0.585–0.738, *P* < 0.001).

## Discussion

In this study, we quantified the dynamic changes in lung and pneumonia on 3D-CT in patients with COVID-19 during hospitalization. The results showed that the lung volume and LAA% significantly decreased in the progressive, peak, and absorption stages. The volume of pneumonia lesions, GGO, and consolidation continually increased in the progressive and peak stages. The GGO/consolidation ratio was significantly reduced in the progressive and peak stages and then recovered a little in the absorption stage. In addition, we found a moderate association between the visual CT score and the volume of pneumonia in the early, progressive, and absorption stages.

The lung is the most involved organ by COVID-19 (18), and typical pathological findings consist of diffuse alveolar epithelium destruction, alveolar septal fibrous proliferation, hyaline membrane formation, and capillary damage/bleeding (19). The pathological changes are the basis of typical CT findings of COVD-19 pneumonia. Previous studies observed the time-dependent changes of COVID-19 pneumonia in the extent and severity of lesions (3, 20, 21), which could be classified as four stages. In the early stage, bilateral GGO with subpleural distribution was the primary manifestation. Subsequently, the infection rapidly progressed to diffuse GGO with crazy-paving pattern, air bronchogram sign, and consolidation. After that, infections in some patients increased to peak involvement in regard to size, number, and density. After the infection was controlled by supportive treatment, the consolidation was absorbed gradually with fibrosis, but GGO appeared due to the absorption of consolidation. Our quantitative analyses perfectly reflected the time course of lung changes on chest CT. We observed a significantly increased volume of pneumonia lesions, GGO, and consolidation in the progressive stage, which slowly increased in the peak stage and significantly decreased in the absorption stage. The mean time to peak of the pneumonia volume was 5.2 ± 4.8 days. The GGO/consolidation ratio was persistently reduced in the progressive and peak stages and then recovered a little in the absorption stage. Timely monitoring the changes of total pneumonia, GGO, and consolidation would be beneficial for the evaluation of treatment response and adjustment of the clinical staging of COVID-19 patients.

The semiquantitative CT score can assess the disease severity of COVID-19 according to the area or degree of lung involvement (3). This marker is simple and readily available in a clinical setting without any need of post-processing. However, it is somewhat subjective when the COVID-19 pneumonia lesions are multiple and irregular. CAD-based quantitative CT might outperform the traditional semiquantitative method based on the visual CT score. Despite the limitation of the CT score, we found a moderate correlation between the visual CT score and the volume of pneumonia measured by our CAD system, indicating that the CT score could be safely and effectively used in clinical practice. Our study showed that the CT scores of progressive and peak stages were significantly higher than those of the early and absorption stages, which may be correlated with elevated inflammation-related biomarkers (22). Yang et al. showed that the CT score was higher in severe cases as compared to mild cases (23). They found that using the CT score of larger than 19.5 could predict severe COVID-19, with high sensitivity (83%) and specificity (94%), which was consistent with the study of Liu et al. (24). Mahdjoub et al. found that the admission CT score was an independent predictor for a 5-day outcome (i.e., mechanical ventilation or death) of COVID-19 patients (25). Therefore, the CT score may help risk stratification and identify those patients at high risk of rapid progress who need timely treatment.

To the best of our knowledge, this study firstly demonstrated that the lung volume of COVID-19 significantly decreased in the progressive and peak stages and then slowly increased in the absorption stage on chest CT, which may suggest the impairment of pulmonary function in COVID-19 patients; but, in the future, a pulmonary function test (PFT) is needed to confirm. Previous studies showed that a significant correlation between lung volume and PFT results in interstitial lung diseases (26–28). Since the outbreak of this disease, its impact on lung function remains unknown. Previous studies have demonstrated that recovered patients with SARS-CoV and MERS-CoV may be left with persistently damaged lung function (11–14). Mo et al. revealed that, in COVID-19 survivors, the most common abnormality of lung function is the reduced diffusion capacity, followed by restrictive ventilatory defect, which is associated with the disease severity (10). A recent case report might suggest that older patients with COVID-19 were more likely to have residual radiological changes and impaired lung function after discharge (29). These preliminary findings might suggest that lung function monitoring and rehabilitation in patients who recovered from COVID-19 is necessary. Our study may provide a possibility to evaluate lung volume without additional health costs.

Our study also has some limitations. First, it is a retrospective study. Second, our selection criteria might have introduced selection bias since we excluded mild and critical illness. Third, the different CT scan parameters might have a potential impact on software-based quantification. Fourth, some patients could not hold well their breath, especially severe cases, which may pose an impact on the calculation. Fifth, the sample size is relatively small, specifically for the peak stage; more subjects are needed to better understand the infection's underlying mechanism and its spread pattern. Sixth, the impact of other patient demographics, such as smoking status and preconditioned respiratory disease wasn't identified, which may play as a confounder. Finally, the effect of other lung structures, such as airways, vessels, and fissures, wasn't observed for analysis.

In summary, quantitative 3D-CT could be used as a useful complementary method to conventional CT in the follow-up of COVID-19 patients. Quantitative assessment of the dynamic changes in lung and pneumonia in patients with COVID-19 may be useful for routine patient management.

## Data Availability Statement

The original contributions generated in the study are included in the article/supplementary material, further inquiries can be directed to the corresponding authors.

## Ethics Statement

The studies involving human participants were reviewed and approved by the Ethics Committee of The First Affiliated Hospital of Jinan University. Written informed consent for participation was not required for this study in accordance with the national legislation and the institutional requirements.

## Author Contributions

QC and LC contributed to the conception and design of the study, the analysis and interpretation of data, and the work drafting. SL, LC, and ML participated in the data extraction and analysis. ZC and JY designed figures. BZ and SZ offered guidance in study design and revised the article critically for important intellectual content. All authors read the revision of the article as well as final approval of the version to be submitted.

## Conflict of Interest

The authors declare that the research was conducted in the absence of any commercial or financial relationships that could be construed as a potential conflict of interest.
